# A Machine Learning Approach to Understanding the Genetic Role in COVID-19 Prognosis: The Influence of Gene Polymorphisms Related to Inflammation, Vitamin D, and *ACE2*

**DOI:** 10.3390/ijms26167975

**Published:** 2025-08-18

**Authors:** Sofía Jaurrieta-Largo, José Pablo Miramontes-González, Luis Corral-Gudino, Miriam Gabella-Martín, Sofía Pérez-Arroyo, Ana M. Torres, Jorge Mateo, José Luis Pérez-Castrillón, Ricardo Usategui-Martín

**Affiliations:** 1 Department of Pneumonology, University Clinical Hospital of Valladolid, 47003 Valladolid, Spain; sofia.jaurrieta@uva.es; 2 Department of Internal Medicine, Río Hortega University Hospital, 47012 Valladolid, Spain; jpmiramontes@uva.es (J.P.M.-G.); luis.corral@uva.es (L.C.-G.); miriam.gabella@uva.es (M.G.-M.); sofia.perez.arroyo@estudiantes.uva.es (S.P.-A.); 3 Department of Medicine, Faculty of Medicine, University of Valladolid, 47003 Valladolid, Spain; 4 Medical Analysis Expert Group, Castilla-La Mancha Institute of Health Research (IDISCAM), 45071 Toledo, Spain; ana.torres@uclm.es (A.M.T.); jorge.mateo@uclm.es (J.M.); 5 Medical Analysis Expert Group, Institute of Technology, University of Castilla-La Mancha, 16071 Cuenca, Spain; 6 Department of Cell Biology, Faculty of Medicine, University of Valladolid, 47005 Valladolid, Spain; 7 Unit of Excellence IOBA, University of Valladolid, 47005 Valladolid, Spain

**Keywords:** COVID-19, genetics, machine learning, inflammation, vitamin D, ACE2

## Abstract

The genetic background influences the outcomes of COVID-19. This study aimed to evaluate the incidence of polymorphisms in genes linked to the RAAS system, cytokine production, and vitamin D on COVID-19 severity, with the goal of gaining a deeper understanding of the genetic etiology related to COVID-19. This study involved 338 COVID-19 patients and employed machine learning methods to identify the genetic variants that most significantly affect COVID-19 severity. The results revealed that polymorphisms in the *IL6*, *IL6R*, *IL1α*, *IL1R*, *IFNγ*, *TNFα*, *CRP*, *VDR*, *VDBP*, and *ACE2* genes are the most significant genetic factors influencing COVID-19 prognosis, particularly in terms of the risks of COVID-19 pneumonia, mortality, rehospitalization, and associated mortality. The machine learning methods achieved an AUC of 0.86 for predicting COVID-19 pneumonia, mortality, and mortality related to rehospitalization, as well as an AUC of 0.85 for rehospitalization within the first year. These results confirm the crucial role of genetic background in COVID-19 prognosis, facilitating the identification of patients at increased risk. In summary, this research demonstrates that genetics-driven machine learning models can pinpoint patients at heightened risk by primarily focusing on genetic variants associated with ACE2, inflammation, and vitamin D.

## 1. Introduction

Coronavirus disease 2019 (COVID-19) is an infectious disease caused by the acute respiratory syndrome coronavirus 2 (SARS-CoV-2) [1]. According to WHO data, the COVID-19 pandemic resulted in 777 million cases and 7 million deaths worldwide [2]. The renin–angiotensin–aldosterone system (RAAS) plays a crucial role in its pathogenesis [3]. SARS-CoV-2 is an angiotensin I-converting enzyme 2 (ACE2) tropic virus; the viral “spike” (S) protein binds to the nasopharyngeal mucosa and alveolar pneumocytes that have ACE2 receptors on their surfaces [4,5]. ACE2, angiotensin II (Ang-II), and Ang 1–7 play crucial roles in regulating fibrosis, inflammation, and thrombosis, thereby modifying edema, permeability, and pulmonary damage [6,7,8]. It has been reported that patients develop a cytokine storm during the progression of the disease. This inflammatory response correlates with the severity of COVID-19 and is characterized by an increase in interleukins (ILs), IFN-γ, TNF-α, and other cytokines. The hyperinflammatory response has also been linked to mortality rates [9,10,11,12]. One of the factors that could modulate the inflammatory response is vitamin D [13,14]. Vitamin D reduces cytokine production to regulate the inflammatory response, which is crucial in respiratory infections, as it helps repair lung epithelial cells [14,15]. In this sense, a correlation has been reported between vitamin D deficiency, thrombotic events, and mortality rates in COVID-19 patients [16,17,18].

The clinical spectrum of COVID-19 ranges from mild to extremely severe cases [1,19]. It has been hypothesized that viral infection drives an exacerbated inflammatory response, leading to severe lung injury that may necessitate hospital admission and mechanical ventilation, increasing the risk of multi-organ failure and death [10]. It has been reported that a genetic etiology is related to the severity of COVID-19. A predisposing genetic background may underlie variations in disease severity among individuals [20,21,22]. Several receptors and metabolic pathways are involved in the pathogenesis of COVID-19 infection. This analysis will consider genes responsible for the renin–angiotensin system, genes that regulate inflammatory cytokines associated with cytokine storms, and genes that determine vitamin D levels and its transport. Using machine learning methods could help identify the genetic variables that have the most significant influence on disease severity, improving the ability to identify high-risk patients. Machine learning fundamentally involves algorithms that take in data, perform computational analysis to predict output values within acceptable accuracy limits, identify patterns and trends, and ultimately learn from past experiences [23,24,25]. Machine learning involves analyzing complex distributions to identify probabilistic associations and the minimal set of features that capture the key patterns in the data, thereby building a predictive model. It has shown better results than traditional, model-based statistical methods [23,24,25].

In this scenario, this study aimed to evaluate the incidence of genetic polymorphisms associated with the RAAS system, cytokine production, and vitamin D on COVID-19 severity, with the goal of gaining a deeper understanding of the genetic etiology related to COVID-19 and developing a machine learning-based risk prediction algorithm that enhances the ability to identify high-risk patients.

## 2. Results

This study involved 338 COVID-19 patients. Table 1 presents the general characteristics of the included patients. A total of 248 patients (73.3%) were diagnosed with COVID-19 pneumonia based on compatible radiographic patterns, and 76 patients (22.4%) passed away during the initial hospitalization. During the first year of clinical follow-up, 77 patients (29.3%) required rehospitalization, while 25 patients died (9.5%). The clinical and analytical variables, as well as the treatments used in the included COVID-19 patients, are summarized in the Supplementary Tables S1–S3. The genotypic distribution of the analyzed single nucleotide polymorphisms (SNPs) according to the risk of COVID-19 pneumonia, mortality, rehospitalization, and mortality related to rehospitalization is shown in Supplementary Table S4.

Table 2 presents various machine learning methods evaluated for predicting the risk of COVID-19 pneumonia, mortality, rehospitalization, and mortality related to rehospitalization based on the genotypic distribution of the polymorphisms. In all cases, XGB was the machine learning method that yielded the best performance for predicting outcomes (Table 2). The XGB method achieved the highest scores across all assessed metrics, including balanced accuracy, recall, precision, area under the curve (AUC), F1 score, Matthews correlation coefficient (MCC), and degenerated Youden index (DYI) kappa. The AUC was 0.86 for predicting COVID-19 pneumonia and mortality, 0.85 for rehospitalization, and 0.86 for mortality associated with rehospitalization (Table 2).

**Figure 1:**
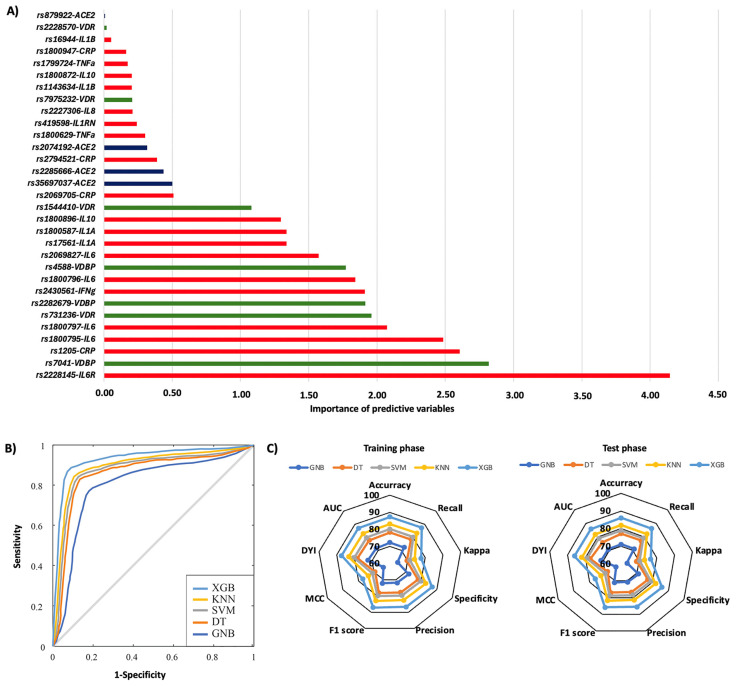
Risk of COVID-19 pneumonia according to genotypic distribution. (**A**) Order of influence of genetic polymorphisms in the risk of COVID-19 pneumonia. The X-axis represents the importance (gain); higher values indicate a greater relative weight in the prediction, without implying direction of effect or relative risk. Red: SNPs are genes related to inflammation, green: SNPs related to vitamin D metabolism, and blue: SNPs in the ACE2 gene. (**B**) ROC curves for the assessed machine learning methods. (**C**) Radar plot in the training phase and the test phase. SVM: Support Vector Machine, DT: Decision Tree, GNB: Gaussian Naïve Bayes, KNN: K-Nearest Neighbors, XGB: Extreme Gradient Boosting.

**Figure 2:**
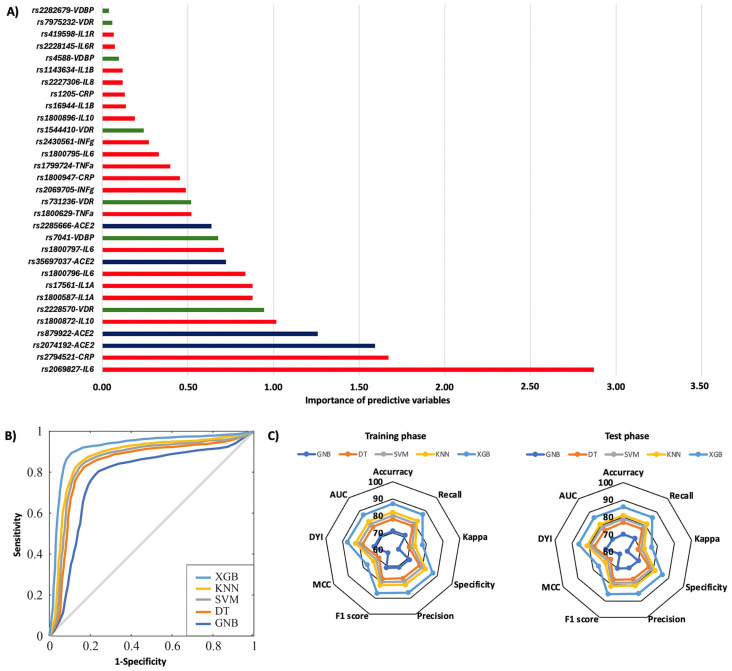
Mortality during the initial hospitalization according to genotypic distribution. (**A**) Order of influence of genetic polymorphisms in the risk of mortality. The X-axis represents the importance (gain); higher values indicate a greater relative weight in the prediction, without implying direction of effect or relative risk. Red: SNPs are genes related to inflammation, green: SNPs related to vitamin D metabolism, and blue: SNPs in the ACE2 gene. (**B**) ROC curves for the assessed machine learning methods. (**C**) Radar plot in the training phase and the test phase. SVM: Support Vector Machine, DT: Decision Tree, GNB: Gaussian Naïve Bayes, KNN: K-Nearest Neighbors, XGB: Extreme Gradient Boosting.

Figure 1 summarizes the risk of COVID-19 pneumonia according to genotypic distribution. Figure 1A illustrates the order of influence of genetic polymorphisms, showing that genetic variants in the *IL6R*, *VDBP*, *CRP*, *IL6*, *VDR*, and *IFN*-γ genes were the most significant in determining the risk of COVID-19 pneumonia. The most influential SNPs were rs2228145 of the *IL6R* gene, particularly the AA genotype, followed by the AA genotype of the rs7041 of the *VDBP* gene. Other crucial polymorphisms included rs1205 in the *CRP* gene, rs1800795 and rs1800797 in the *IL6* gene, rs731236 in the *VDR* gene, rs2282679 in the *VDBP* gene, and rs2430661 in the *IFN-γ* gene. The ROC curve was calculated, and the results indicated that the system based on the XGB model achieves a larger area, enabling greater accuracy in predicting the risk of COVID-19 pneumonia (Figure 1B). The AUC was 0.86. The radar plots indicated that the model training subsets resembled the scores in the test subsets. The XGB system exhibited a larger area (Figure 1C). The TT genotype of the rs2069827 polymorphism in the *IL6* gene was the most significant genetic variant in predicting the risk of mortality caused by COVID-19. The second most influential factor was the CC genotype of the *CRP* rs2794521 polymorphism. Also crucial were the rs2074192, rs2074192, rs35697037, and rs2285666 polymorphisms in the *ACE2* gene; rs1800872 in *IL10*; rs2228570 in the *VDR* gene; rs1800587 and rs17561 in the *IL1A* gene; rs1800796 and rs1800797 in the *IL6* gene; and rs7041 in the *VDBP* gene (Figure 2A). The ROC curve showed that the XGB model system has a larger area, thereby enhancing accuracy in predicting mortality caused by COVID-19, with an AUC of 0.86 (Figure 2B). The XGB model also exhibited a larger area in the radar plots for both the training and test subsets (Figure 2C).

**Figure 3:**
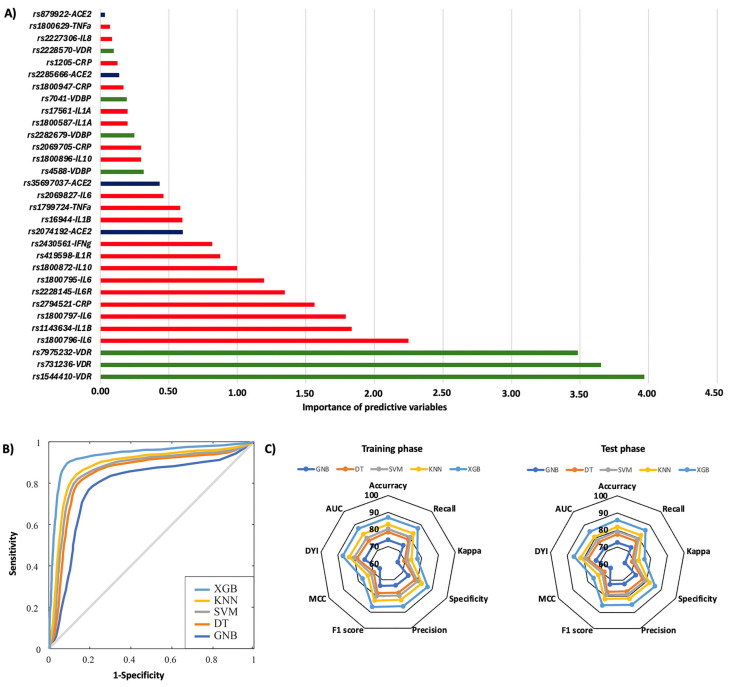
Risk of rehospitalization according to genotypic distribution. (**A**) Order of influence of genetic polymorphisms in the risk of rehospitalization. The X-axis represents the importance (gain); higher values indicate a greater relative weight in the prediction, without implying direction of effect or relative risk. Red: SNPs are genes related to inflammation, green: SNPs related to vitamin D metabolism, and blue: SNPs in the ACE2 gene. (**B**) ROC curves for the assessed machine learning methods. (**C**) Radar plot in the training phase and the test phase. SVM: Support Vector Machine, DT: Decision Tree, GNB: Gaussian Naïve Bayes, KNN: K-Nearest Neighbors, XGB: Extreme Gradient Boosting.

**Figure 4:**
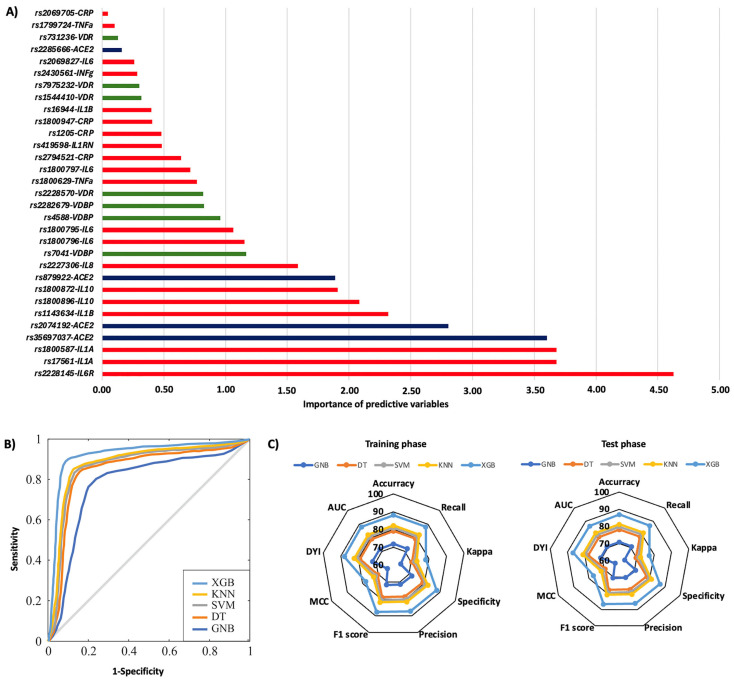
Risk of mortality on rehospitalization according to genotypic distribution. (**A**) Order of influence of genetic polymorphisms in the risk of mortality on rehospitalization. The X-axis represents the importance (gain); higher values indicate a greater relative weight in the prediction, without implying direction of effect or relative risk. Red: SNPs are genes related to inflammation, green: SNPs related to vitamin D metabolism, and blue: SNPs in the ACE2 gene. (**B**) ROC curves for the assessed machine learning methods. (**C**) Radar plot in the training phase and the test phase. SVM: Support Vector Machine, DT: Decision Tree, GNB: Gaussian Naïve Bayes, KNN: K-Nearest Neighbors, XGB: Extreme Gradient Boosting.

In the first year, 29.3% of patients were rehospitalized, and among them, 32.4% died. The rs1544410, rs731236, and rs7975232 polymorphisms in the *VDR* gene were the most influential factors affecting the risk of rehospitalization, particularly the TT, AA, and AA genotypes, respectively (Figure 3A). Additionally, significant SNPs were identified in the *IL6* gene (rs1800796, rs1800797, and rs1800795), the *IL1B* gene (rs1143634), *CRP* (rs2294521), and the *IL6R* gene (rs2228145) (Figure 3A). The AA genotype of the *IL6R* rs2228145 polymorphism was most significantly linked to mortality related to rehospitalization (Figure 4A). Polymorphisms in the *IL1A* (rs17561 and rs1800587), *ACE2* (rs35697037, rs2074192, and rs879922), *IL1B* (rs1143634), *IL10* (rs1800896 and rs1800872), and *IL8* (rs2227306) genes were also crucial genetic factors for predicting mortality associated with rehospitalization in the first year following the first COVID-19 diagnosis (Figure 4A). The XGB model was the best for predicting rehospitalization and associated mortality, demonstrating a larger ROC curve area (AUC was 0.85 for rehospitalization and 0.86 for mortality related to rehospitalization) (Figure 3B and Figure 4B). Furthermore, the XGB model produced the largest area in the training and test subsets (Figure 3C and Figure 4C).

## 3. Discussion

The COVID-19 outcomes range from mild to severe, and the severity of the disease may partly depend on genetic background [1,20]. This study employs a machine learning methodology to identify the genetic variants that most significantly influence COVID-19 severity and to develop a genetically based risk prediction algorithm that enhances the ability to identify high-risk patients. In this context, our results identified polymorphisms in genes such as *IL6*, *IL6R*, *IL1A*, *IL1R*, *IFN*-γ, TNF-α, *CRP*, *VDR*, *VDBP*, and *ACE2* as the most significant genetic factors influencing COVID-19 prognosis, especially regarding the risks of COVID-19 pneumonia, mortality, rehospitalization, and related mortality. The machine learning methods yielded an AUC of 0.86 for predicting COVID-19 pneumonia, mortality, and rehospitalization-associated mortality, as well as an AUC of 0.85 for rehospitalization within the first year. These results confirm the crucial role of the genetic background in COVID-19 prognosis, enabling the identification of patients at increased risk. Nineteen genetic variants in ten different genes were the most influential in COVID-19 prognosis, particularly *IL6R* (rs2228145), *IL6* (rs1800797 and rs1800796), *CRP* (rs2794521 and rs1800947), *IFN*-γ (rs2430561), *IL1A* (rs17561 and rs1800587), *TNF-α* (rs1800629), *IL1R* (rs419598), *ACE2* (rs35697037 and rs2285666), *VDR* (rs1544410, rs7975232, and rs2228570), and *VDBP* (rs7041, rs2282679, and rs4588). These results highlight the crucial role of gene polymorphisms in inflammation, vitamin D, and the *ACE2* in COVID-19 outcomes.

Disease severity varies, ranging from asymptomatic to patients who require intensive care unit admission and mechanical ventilation. Factors such as age, multiple comorbidities, and sex have been linked to COVID-19 [1]. This variability has also been associated with genetic factors; SNPs in specific genes could impact the variation in the clinical spectrum of COVID-19 [26,27]. Principally, it has focused on the role of genetic factors in the disease’s progression and severity, including polymorphisms in *ACE*, *IL6*, *TNF-α,* or *VDR* genes [27]. Our results reinforce the critical importance of genetic background in COVID-19 pathogenesis, particularly genetic variants related to *ACE2*, inflammation, and vitamin D. SARS-CoV-2 enters epithelial cells by binding to the ACE2 receptor. Furthermore, ACE2 regulates fibrosis, inflammation, and thrombosis, all of which impact edema, permeability, and lung damage [6,7,8]. Thus, multiple genetic polymorphisms in *ACE2* that alter the structure or the expression rate have been linked to COVID-19 susceptibility and severity [26,27,28,29,30]. Using machine learning methods, this study demonstrated that the rs35697037 and rs2285666 polymorphisms were the most significant SNPs in determining COVID-19 outcomes, particularly hospitalization and mortality, related to the *ACE2* gene.

SARS-CoV-2 infection activates both innate immunity, with alveolar macrophages playing a key role, and adaptive immunity to prevent proliferation. However, the virus has mechanisms to bypass these defense systems, which can lead to tissue damage and the recruitment of immune cells responsible for the cytokine storm that may persist after viral clearance. The body’s inability to control this response, which may be genetically mediated, can result in poor disease outcomes [31]. The principal pathological mechanism in COVID-19 is the excessive release of cytokines, known as a cytokine storm, which leads to lung injury and multi-organ damage [32]. In this scenario, multiple studies have linked polymorphisms in inflammatory-related genes to disease severity, particularly genetic variants that alter cytokine expression [26,27,33,34]. Our results with machine learning methods confirm their importance in patient outcomes. Additionally, our results underscore the crucial role of SNPs in the *CRP* gene. The CRP concentrations have been previously associated with disease progression [35], but few studies are exploring the impact of SNPs in the *CRP* gene on the severity of COVID-19.

Vitamin D, aside from its effects on bone metabolism, regulates the expression of genes involved in various biological functions, including organ development, cell cycle control, phosphocalcic metabolism, detoxification, and the regulation of innate and adaptive immunity [36]. One hallmark of vitamin D’s effects is the regulation of genes involved in inflammatory processes. Accordingly, there is an interplay between vitamin D signaling and other signaling cascades that are involved in inflammation [37]. The rapid increase in the serum 25(OH)D3 concentrations was related to a decrease in innate immunity markers, including eotaxin, IL12, monocyte chemoattractant protein-1, and macrophage inflammatory protein-1beta. Vitamin D metabolism is enzymatically regulated and depends on polymorphisms in multiple genes involved [38]. Mainly, polymorphisms have been described in genes such as *CYP2R1* [39], *GC* (which encodes the transporter protein DBP) [40], *CYP24R* [41], *DHCR7* [42], and *VDR* (vitamin D receptor) [43]. In this scenario, polymorphisms influencing vitamin D activity, such as those in the *VDR* and *VDBP* genes, have also been associated with COVID-19 infection and its clinical progression [27,44,45,46,47]. Our results demonstrated the significant role of genetic variants in *VDR* and *VDBP* in the risk of COVID-19 pneumonia, mortality, and rehospitalization, confirming that vitamin D metabolism is essential in the pathology of COVID-19.

This study highlights the importance of genetic background in determining the severity of COVID-19. The results highlight the significance of polymorphisms in *ACE2*, inflammatory, and vitamin D genes in determining the risk of COVID-19 pneumonia, mortality, rehospitalization, and related mortality. This research also presents machine learning methods as a tool that allows the construction of genetic-based models to identify patients with a worse prognosis. Our results involved predictive algorithms that combined the influence of several SNPs. The models obtained have an AUC greater than 0.85, indicating the remarkable predictive power of the models for identifying COVID-19 outcomes and patients at increased risk. The main limitation of our study is that the cohort size did not permit a more detailed analysis. Additional studies in different patient series would be necessary. In addition, to delve deeper into this topic, functional studies in molecular and cellular biology would need to be developed. Nonetheless, this study provides, for the first time, a genetically based machine learning model to identify COVID-19 severity. Additionally, this research lays the groundwork for future studies to incorporate a broader range of patients and develop more precise and effective algorithms for predicting COVID-19 outcomes. In conclusion, this study highlights that genetics-based machine learning models can identify patients with an increased risk, primarily considering genetic variants in *ACE2*, inflammation, and vitamin D.

## 4. Materials and Methods

### 4.1. Patients

Consecutive patients with COVID-19 who developed acute respiratory distress syndrome (ARDS) were included in this study. The patients were diagnosed in the Río Hortega University Clinical Hospital (Valladolid, Spain) between March and November 2020. The primary inclusion criteria were patients over 18 years of age with a microbiological diagnosis of COVID-19, symptoms of lung involvement, an admission radiological image compatible with the diagnosis, and laboratory and clinical variables upon admission, as well as signing the informed consent form. The radiological images were classified into three groups according to Litmanovich et al. [48]: (1) typical presentation of COVID-19 pneumonia; (2) indeterminate, less typical findings of COVID-19 pneumonia that may occur in a variety of infectious and non-infectious processes; and (3) atypical or uncommon findings of COVID-19 pneumonia, making it necessary to consider alternative diagnoses. Exclusion criteria included the following: age under 18 years, diagnosis of active tumor disease, and patients with other diseases with a life expectancy of less than 6 months. Patients with atypical or uncommon findings in radiological images were also excluded. Furthermore, the fact that the informed consent form was not signed was another exclusion criterion.

Demographic variables, medical history, and previous pharmacological and non-pharmacological treatments were collected, along with laboratory parameters including complete blood count, biochemistry, coagulation profile, and inflammatory markers. Clinical features at the onset of infection, complications, and progression during hospitalization were also collected, including pharmacological and non-pharmacological treatments received during the hospital admission. Study participants were clinically followed up for a year. Additionally, venous blood samples were collected in tubes containing EDTA during hospital admission. This study employed a double-masked (blinded) design to minimize bias.

### 4.2. DNA Isolation and Polymorphism Genotyping

Venous blood samples were collected in EDTA tubes, and the genomic DNA was extracted from peripheral blood leukocytes using the Purelink Genomic DNA Mini Kit (Invitrogen, Paisley, UK). DNA was quantified and diluted to a final concentration of 100 ng/μL. Genotyping was performed using TaqMan 5’-exonuclease allelic discrimination assays that contain sequence-specific forward and reverse primers to amplify the polymorphic sequences and two probes labeled with VIC and FAM dyes to detect both alleles of each polymorphism [49]. PCR reactions were performed using TaqMan Universal PCR Master Mix according to the manufacturer’s instructions in a StepOne Plus Real-Time PCR system (Thermofisher, Applied Biosystems, MA, USA). To assess reproducibility, a random selection of 5% of the samples was re-genotyped, and all of these genotypes matched the initially obtained genotypes. It analyzed the genotypic distribution of SNPs in genes involved in inflammatory activation, vitamin D metabolism, and the RAAS system. The genotypic distribution of the SNPs in the 338 COVID-19 patients is shown in Supplementary Table S4. Genetic polymorphisms were selected according to the following considerations: (1) functionality (previously described or possible effect) and (2) distribution along the gene, with preference given to those located in exons or contiguous regions. The Genecard (www.genecards.org, accessed on 4 March 2021) and NCBI (www.ncbi.nlm.nih.gov/snp, accessed on 4 March 2021) databases were utilized to identify the genes and pathways associated with each included polymorphism.

### 4.3. Machine Learning Analysis

Machine learning methods were employed to analyze the associations between genetic distributions of polymorphisms, COVID-19 pneumonia (diagnosed by compatible radiographic patterns), and the risk of death during the initial hospitalization. Additionally, the patients were clinically followed for one year and assessed for rehospitalization and risk of death related to COVID-19. The XGB method was proposed as the primary approach for data analysis due to its scalability, rapid execution, and impressive accuracy. Additionally, its versatility facilitates parallel computing [50]. Other machine learning methods in the literature have assessed the efficacy and performance of this system. They were the Support Vector Machine (SVM) [51], Decision Tree (DT) [52], Gaussian Naïve Bayes (GNB) [53], and K-Nearest Neighbors (KNN) [54]. Models derived from these methodologies were created using MATLAB v2 (The MathWorks, Natick, MA, USA; MATLAB R2023) [55].

Supplementary Figure S1 summarizes the steps taken to implement the machine learning algorithms. This research employed nested cross-validation in conjunction with Bayesian optimization techniques to effectively and reliably tune the hyperparameters of machine learning models. In the nested cross-validation process, the outer loop assessed the model’s overall performance while the inner loop concentrated on optimizing hyperparameters. Bayesian optimization was utilized in the inner loop to effectively navigate the space of critical hyperparameters, such as maximum tree depth (max_depth), number of estimators (n_estimators), learning rate (learning_rate), and regularization terms (lambda and alpha). Bayesian optimization employed a probabilistic model rooted in a Gaussian process to uncover optimal hyperparameter combinations. This approach utilized insights from prior iterations, reducing the necessity for exhaustive evaluations and concentrating on the most promising areas [56,57]. This method mitigated the risk of overfitting by keeping the test data in the outer loop separate from the optimization process. It enhanced model stability through uniform evaluations over various data partitions. The synergy of these strategies yielded models with improved performance and robust generalizability. Drawing from a systematic feature relevance analysis, we implemented a hybrid approach to identify the most impactful variables. We first assessed feature importance using a preliminary XGB model, which generated scores based on gain, coverage, or weight. This was followed by iterative feature selection methods, including Recursive Feature Elimination (RFE) with XGBoost as the base estimator, which aimed to trim the feature set while preserving strong predictive performance. Finally, we evaluated the effect of omitting specific features through cross-validation, ensuring that only those significantly enhancing the model’s performance were retained.

To minimize overfitting in XGBoost, we implemented several techniques. These included using explicit regularization through the lambda and alpha parameters, controlling the maximum tree size (max_depth), lowering the learning rate (learning_rate), and applying early stopping to end training if validation metrics did not improve after several consecutive iterations. Bootstrap validation was conducted, creating various data subsets via resampling to assess uncertainty in performance metrics and guarantee consistency. Furthermore, the model was validated on an independent external cohort to evaluate its generalizability across diverse clinical settings. The data were randomly split into training (70%) and testing (30%) sets to ensure a balanced representation of key classes and characteristics. The simulations were rigorously executed over 100 iterations, carefully accounting for mean and standard deviation values, thus reducing the potential impact of noise and ensuring the achievement of statistically valid conclusions [58].

### 4.4. Ethical Aspects

This study involving human subjects was conducted following the tenets of the Declaration of Helsinki (2008) and received approval from the Ethics Committee of Río Hortega University Hospital in Valladolid (PI216-20, approval date: 29 May 2020 and 2 March 2021). This study fully complied with the ethical standards of the World Medical Association, as well as Spanish data protection laws (LO 15/1999) and related regulations (RD 1720/2007). All patients who agreed to participate provided signed written consent.

## Figures and Tables

**Table 1:** General characteristics of the study cohort.

General Characteristics	Patients
Age, mean (SD) (years)	73.26 (13.09)
Sex (male; female), n (%)	182 (53.84); 156 (46.15)
Days from symptom onset to hospital admission, mean (SD)	5.77 (5.45)
COVID-19 pneumonia, n (%)	248 (73.3)
Days of admission, mean (SD)	18.21 (22.75)
Death due to COVID-19, n (%)	76 (22.48)
Dependency (yes; moderate; mild; independent), n (%)	42 (12.42); 47 (13.90); 169 (50); 80 (83.67)
Rehospitalization in the first year, n (%)	77 (29.3)
Smoking history (active; ex-smoker; never smoker), n (%)	12 (3.56); 57 (16.91); 268 (79.52)
Dementia, n (%)	41 (12.20)
Hypertension, n (%)	196 (58.16)
Dyslipidemia n (%)	133 (39.58)
Myocardial infarction (%)	15 (4.45)
Heart failure, n (%)	20 (5.97)
Cerebral ictus, n (%)	18 (5.36)
Diabetes mellitus, n (%)	68 (20.24)
COPD, n (%)	8 (2.43)
Asthma, n (%)	32 (9.46)
Obstructive sleep apnea, n (%)	20 (5.93)
Chronic kidney disease (4–5), n (%)	26 (7.15)
Tumor without metastasis, n (%)	39 (11.57)
Tumor with metastasis, n (%)	5 (1.48)
ACEi, n (%)	82 (24.40)
ARBs, n (%)	77 (22.91)
Statins, n (%)	81 (24.11)
Metformin, n (%)	36 (10.71)
DDP-4 inhibitors, n (%)	30 (8.92)
Insulin, n (%)	20 (5.95)
Inhaled corticosteroids, n (%)	33 (9.82)
Corticosteroids, n (%)	10 (2.97)
Immunosuppressors/immunomodulators, n (%)	16 (4.76)
Test COVID-19 confirmation, n (%)	338 (100)

**Table 2:** Different machine learning methods were tested to predict the risk of COVID-19 pneumonia, mortality, rehospitalization, and mortality on rehospitalization according to the genotypic distribution of the polymorphisms in patients included in this study.

	Method	BA (%)	Recall	Precision	AUC	F1 Score	MCC	DYI	Kappa
COVID-19 pneumonia	SVM	79.09	79.18	78.53	0.79	78.85	70.18	79.09	70.41
	DT	77.23	77.32	76.68	0.76	77.00	68.53	77.23	68.75
	GNB	71.16	71.25	70.65	0.71	70.95	63.14	71.16	63.35
	KNN	82.04	82.13	81.45	0.82	81.79	72.79	82.04	73.04
	XGB	86.10	86.20	85.48	0.86	85.84	76.40	86.10	76.65
Mortality	SVM	79.20	79.29	78.64	0.79	78.96	70.28	79.20	70.51
	DT	77.13	77.22	76.58	0.76	76.90	68.44	77.13	68.67
	GNB	70.18	70.26	69.68	0.70	69.97	62.27	70.18	62.48
	KNN	81.17	81.27	80.59	0.81	80.93	72.02	81.17	72.26
	XGB	86.00	86.10	85.39	0.86	85.74	76.31	86.00	76.56
Rehospitalization	SVM	79.26	79.35	78.69	0.78	79.02	70.33	79.26	70.56
	DT	77.48	77.58	76.93	0.77	77.25	68.75	77.48	68.98
	GNB	72.78	72.86	72.25	0.72	72.56	64.46	72.78	64.68
	KNN	81.94	82.04	81.36	0.81	81.69	72.71	81.94	72.95
	XGB	85.86	85.96	85.25	0.85	85.61	76.19	85.86	76.44
Mortality (rehospitalization)	SVM	79.85	79.94	79.28	0.80	79.61	70.85	79.85	71.09
	DT	78.12	78.21	77.56	0.78	77.88	69.31	78.12	69.54
	GNB	70.83	70.91	70.32	0.71	70.62	62.85	70.83	63.05
	KNN	81.17	81.27	80.59	0.81	80.93	72.02	81.17	72.26
	XGB	86.85	86.72	86.23	0.86	86.59	77.06	86.85	77.32

BA: Balanced Accuracy. AUC: Area Under Curve. MCC: Matthew Correlation Coefficient. DYI: Degenerated Younden Index. SVM: Support Vector Machine. DT: Decision Tree. GNB: Gaussian Naïve Bayes. KNN: K-Nearest Neighbors. XGB: Extreme Gradient Boosting.

## Data Availability

All data needed to evaluate the conclusions in this paper are present in this paper and/or the Supplementary Materials. Additional data related to this paper may be requested from the authors.

## Supplementary Materials

The following supporting information can be downloaded at: https://www.mdpi.com/article/10.3390/ijms26167975/s1.
