# Supplementary material for: A Machine Learning Approach to Understanding the Genetic Role in COVID-19 Prognosis: The Influence of Gene Polymorphisms Related to Inflammation, Vitamin D, and ACE2
Source: Int J Mol Sci. 2025 Aug 18;26(16):7975. doi: 10.3390/ijms26167975 (PMC12387086; doi:10.3390/ijms26167975)
Supplement: Supplementary file 1 [file ijms-26-07975-s001.zip › ijms-3757990-supplementary.pdf]

Supplementary Materials

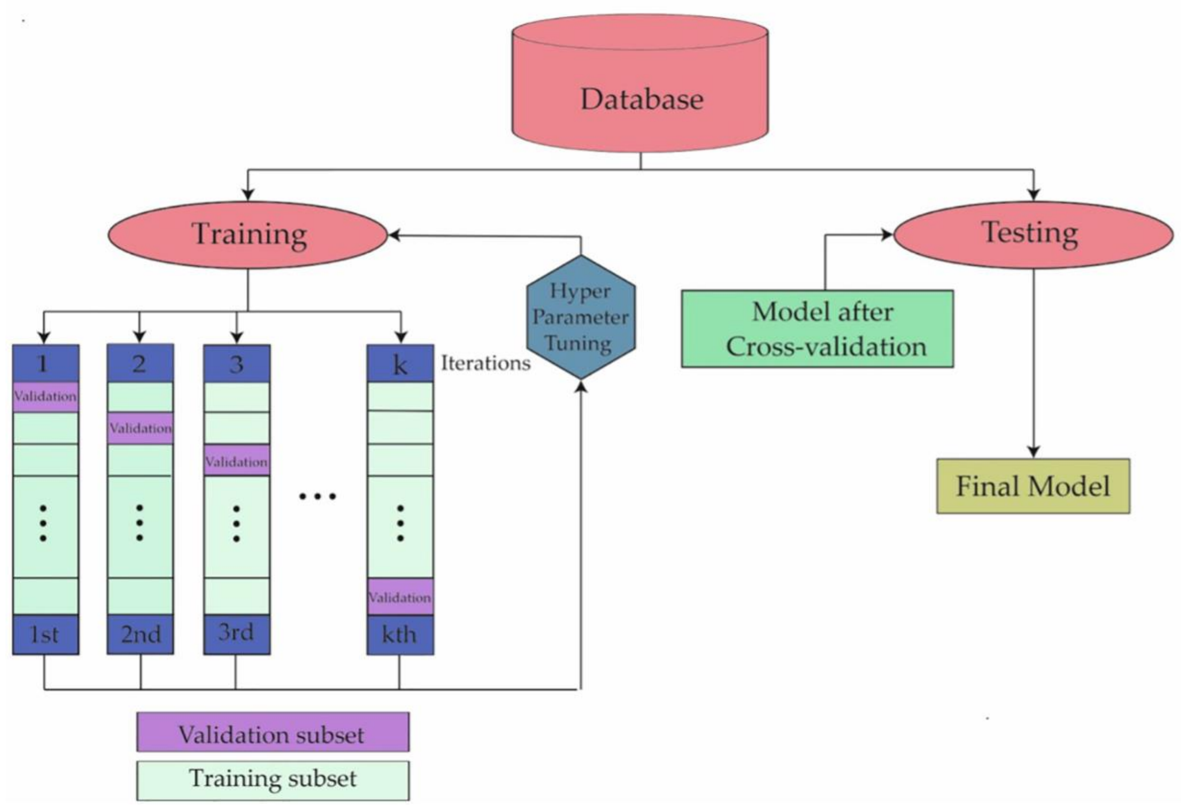

Supplementary Figure S1. Steps taken to implement the machine learning algorithms.

Supplementary Table S1. Clinical variables associated with COVID-19 disease.

Clinical Variable	Patients
Dyspnoea, n(%)	180(53.57)
Fever, n(%)	209(62.20)
Low grade fever, n(%)	262(77.97)
Cough, n(%)	175(51.93)
Diarrhoea, n(%)	74(21.96)
Sickness, n(%)	45(13.35)
Anorexia, n(%)	45(13.35)
Abdominal pain, n(%)	23(6.85)
Asthenia, n(%)	111(33.04)
Arthralgia, n(%)	33(9.82)
Anosmia, n(%)	20(5.93)
Ageusia, n(%)	22(6.55)
Pharyngitis, n(%)	14(4.17)
Headache, n(%)	28(8.31)
ARDS, n(%)	61(18.94)
Hyperinflammatory data in laboratory results, n(%)	126(39.13)
Pulmonary embolism/Deep vein thrombosis, n(%)	5(1.55)
Septic shock, n(%)	15(4.66)

Supplementary Table S2. Analytical variables of the COVID-19 patients included.

Analytical Variables	Patients	Refrerence Values
PaO2, mean (SD) (mmHg)	73.98 (36.4)	83–108
Leukocytes, mean(SD) (x106/l)	6.843 (3100)	4–10.5
Lymphocytes, mean (SD)(x106/l)	1.007 (581)	0.9–5.2
Platelet, mean (SD)(x109/l)	201.523 (93.746)	150.000–400.000
Haemoglobin, mean (SD) (g/dl)	12.74 (2.11)	12–18
D- dimer, mean (SD) (μg/l)	1889,25 (4801.7)	0–500
PCR, mean (SD) (mgr/l)	103.05 (88.73)	0–10
Ferritin, mean (SD) (μgr/l)	763.40 (801.43)	20–300
LDH, mean (SD)(UI/l)	342.09 (147.53)	110–210
Creatinine, mean (SD) (mg/dl)	1.88 (11.18)	0.8–1.3
Sodium, mean (SD)(mEq/l)	136.52 (5.33)	136–146
GPT, mean (SD)(UI/l)	35.81 (29.57)	1–50
GOT, mean (SD) (UI/l)	44.81 (25.91)	0–50
IL-6, mean (SD) (pg/ml)	31.05 (17.28)	<7
SAFI (SpO2/FiO2) %,mean (SD)	424.22 (68.51)	>400
PAFI (PaO2/FiO2) %, mean (SD)	282.39 (87.77)	>300

Supplementary Table S3. Treatments of the COVID-19 patients included.

Treatments	Patients
Antibiotic, n(%)	314 (97.82)
Hydroxychloroquine, n(%)	268 (84.27)
Lopinavir/Ritonavir, n(%)	189 (59.43)
Interferon, n(%)	0 (0)
Remdesivir, n(%)	0 (0)
Anticoagulation, n(%)	229 (71.56)
Tocilizumab, n(%)	14 (4.42)
Glucocorticoids, n(%)	98 (30.62)
Nasal cannula/Ventimask/oxygen reservoir n(%)	186 (57.76); 68 (21.12); 68 (21.12)
High-flow nasal cannula, n(%)	24 (7.47)
Non-invasive mechanical ventilation, n(%)	38 (11.84)
Invasive mechanical ventilation, n(%)	43 (13.4)
Extracorporeal Membrane Oxygenation (ECMO), n(%)	7 (2.17)

Supplementary Table S4. Genotypic distribution according to the risk of COVID-19 pneumonia, mortality, re-hospitalization, and mortality related to re-hospitalization.

	Genotypic Distribution, n (%)				
SNP	All COVID-19 Patients, n (%)	COVID-19 Pneumonia, n (%)	Mortality, n (%)	Re-Hospitalization, n (%)	Mortality Related to Re-Hospitalization, n (%)
**rs1544410 (CC/CT/TT)**	131(38.8) / 151(44.7) / 56(16.6)	88(35.5) / 117(47.2) / 43(17.3)	29(38.2) / 29(38.2) / 18 (23.7)	36(46.8%) / 38(49.4) / 3(3.9)	12 (48.0) / 11 (44.0) / 2(8%)
**rs1800795 (CC/CG/GG)**	34(10.1) / 156(46.2) / 148(43.8)	26(10.5) / 119(48.0) / 103(41.5)	4(5.3) / 43(56.6) / 29(38.2)	12(15.6) / 32(41.6%) / 33(42.9)	6(24.0) / 10(40.0) / 9(36.0)
**rs2069827 (GG/GT/TT)**	300(88.8) / 18(5.3) / 20(5.9)	218(87.9) / 29(11.7) / 1(0.4)	72(94.7%) / 4(5.3)/ (-)	68(88.3) / 8(10.4) / 1(1.3)	22(88.0) / 3(12.0) / (-)
**rs2074192 (CC/CT/TT)**	163(48.2) / 78(23.1) / 97(28.7)	117(47.2) / 54(21.8) / 77(31.0)	36(47.4) / 17(22.4) / 23(30.3)	41(53.2) / 15(19.5) / 21(27.3)	10(40.0) / 6(24.0) / 9(36.0)
**rs2228570 (GG/GA/AA)**	153(45.3) / 147(43.5) 36 (10.7)	113(45.9) / 106(43.1) / 27(11.0)	35(46.7) / 34(45.3) / 6(8.0)	33(42.9) / 34(44.2) / 10(13.0)	10(40.0) / 14(56.0) / 1(4.0

**rs2285666 (TT/TC/CC)**	44(13.0) / 54(16.0) / 240(71.0)	30(12.1) / 35(14.1) / 183(73.8)	9(11.8) / 12(15.8%) / 55(72.4)	12(15.6) / 11(14.3) / 54(70.1)	4(16.0) / 5(20.0) / 16(64.0)
**rs731236 (GG/GA/AA)**	59(175) / 145(42.9) / 133(39.3)	45(18.2) / 116(47.0) / 86(34.8)	20(26.3) / 26(34.2) / 30(39.5)	4(5.2) / 36(46.8) / 37(48.1)	2(8.0) / 11(44.0) / 12(48.0)
**rs7975232 (CC/CA/AA)**	71(21.0) / 166(49.1) / 101(29.9)	45(18.1) / 128(51.6) / 75(30.2)	17(22.4) / 34(44.7) / 25(32.9)	20(26.0) / 42(54.5) / 15(19.5)	5(20.0) / 15(60.0) / 5(20.0)
**rs879922 (CC/CG/GG)**	103(30.5) / 60(17.8) / 174(51.5)	77(31.2) / 38(15.4) / 132(53.4)	24(31.6) / 11(14.5) / 41(53.9)	26(34.2) / 13(17.1) / 37(48.7)	6(24.0) / 3(12.0) / 16(64.0)
**rs1059047 (TT/TC/CC)**	295(87.3) / 20 (5.9) / 21(6.8)	214(87.0) / 32(13.0) / (-)	65(85.5) / 11(14.5) / (-)	69(89.6) / 7(9.1) / 1(1.3)	22(88.0) / 2(8.0) / 1(4.0)
**rs111521887 (CC/CG/GG)**	246(72.8) / 79(23.4) / 8(2.4)	187(76.6) / 53(21.7) / (-)	49(66.2) / 23(31.1) / 2(2.7)	56(73.7) / 19(25.0) / 1(1.3)	18(72.0) / 5(20.0) / 2(8.0)
**rs1143634 (GG/GA/AA)**	210(62.1) / 112(33.1) / 14(4.1)	151(61.4) / 84(34.1) / 11(4.5)	45(59.2) / 27(35.5) / 4(5.3)	44(57.1) / 25(32.5) / 8(10.4)	18(72.0) / 6(24.0) 1(4)
**rs1205 (TT/TC/CC)**	37(10.9) / 148 (43.8) /151(44.7)	26(10.6) / 117(47.6) / 103(41.9)	7(9.2) / 37(48.7) / 32(42.1)	10(13.0) / 29(37.7) / 38(49.4)	1(4.0) / 11(44.0) / 13(52.0)
**rs1544410 (TT/TC/CC)**	55(16.3) / 150(44.4) / 131(38.8)	42(17.1) / 116(47.2) / 88(35.8)	18(23.7) / 29(38.2) / 29(38.2)	3(3.9) / 38(49.4) / 36(46.8)	2(8.0) / 11(44.0) / 12(48.0)
**rs16944 (GG/GA/AA)**	164(48.5) / 143(42.3) / 29(8.6)	120(48.8) / 104(42.3) / 22(8.9)	37(48.7) / 33(43.4) / 6(7.9)	39(50.6) / 32(41.6) / 6(7.8)	12(48.0) / 9(36.0) / 4(16.0)
**rs17561 (CC/CA/AA)**	190(56.2) / 121(35.8) / 25(7.4)	136(55.3) / 91(37.0) / 19(7.7)	42(55.3) / 28(36.8) / 6(7.9)	42(54.5) / 25(32.5) / 10(13.0)	19(76.0) / 4(16.0) / 2(8.0)
**rs17690703 (CC/CT/TT)**	166(49.1) / 139(41.1) / 31(9.2)	131(53.3) / 95(38.6) / 20(8.1)	37(48.7) / 32(42.1) / 7(9.2)	38(49.4) / 30(39.0) / 9(11.7)	9(36.0) / 15(60.0) / 1(4.0)
**rs1799724 (TT/TC/CC)**	5(1.5) / 68 (20.1) / 263 (77.8)	3(1.2) / 50(20.3) / 193(78.5)	2(2.6) / 13(17.1) / 61(80.3)	2(2.6) / 15(19.5) / 60(77.9)	1(4.0) / 5(20) / 19(76.0)
**rs1800587 (AA/AG/GG)**	25(7.4) / 121(35.8) / 190(56.2)	19(7.7) / 91(37.0) / 136(55.3)	6(7.9) / 28(36.8) / 42(55.3)	10(13.0) / 25(32.5) / 42(54.5)	2(8.0) / 4(16.0) / 19(76.0)
**rs1800629 (AA/AG/GG)**	9(2.7) / 60(17.8) / 267(79.0)	6(2.4) / 46(18.7) / 194(78.9)	3(3.9) / 17(22.4) / 56(73.7)	3(3.9) / 13(16.9) / 61(79.2)	(-) / 4(16.0) / 21(84.0)
**rs1800796 (CC/CG/GG)**	8(2.4) / 43(12.7) / 285(84.3)	6(2.4) / 34(13.8) / 206(83.7)	1(1.3) / 10(12.2) / 65(85.5)	1(1.3) / 10(13.0) / 66(85.7)	2(8.0) / 3(12.0) / 20(80.0)
**rs1800797 (AA/AG/GG)**	34(10.1) / 156(46.2) / 146(43.2)	25(10.2) / 120(48.8) / 101(41.1)	5(6.6) / 43(56.6) / 28(36.8)	12(15.6) / 33(42.9%) / 32(41.6)	6(24.0) / 9(36.0) / 9(36.0)
**rs1800872 (GG/GT/TT)**	173(51.2) / 132(39.1) / 31(9.2)	130(52.8) / 97(39.4) / 19(7.7)	41(53.9) / 28(36.8) / 7(9.2)	46(59.7) / 22(28.6) / 9(11.7)	10(40.0) / 9(36.0) / 6(24.0)
**rs1800896 (CC/CT/TT)**	51(15.1) / 154(45.6) / 131(38.8)	40(16.3) / 114(46.3) / 92(37.4)	14(18.4) / 30(39.5) / 32(42.1)	13(16.9) / 35(45.5) / 29(37.7)	(-) / 13(52.0) / 12(48.0)
**rs1800947 (CC/CG/GG)**	301(89.1) / 34(10.1) / 1(0.3)	223(90.7) / 22(8.9) / 1(0.4)	67(88.2) / 9(11.8) / (-)	68(88.3) / 8(10.4) / 1(1.3)	23(92.0)/ 2(8.0) / (-)
**rs1884082 (GG/GT/TT)**	102(30.2) / 157(46.4) / 76(22.5)	77(31.4) / 111(45.3) / 57(23.3)	21(27.6) / 35(46.1) / 20(26.3)	24(31.2) / 37(48.1) / 16(20.8)	5(20.0) / 14(56.0) / 6(24.0)
**rs199422297 (GG/GA/AA)**	336(100) / (-) /(-)	246 (100) / (-) / (-)	76(100) / (-) / (-)	77(100) / (-) / (-)	25(100) / (-) / (-)
**rs2069705 (GG/GA/AA)**	31(9.2) / 141(41.7) / 163(48.2)	23(9.4) / 102(41.6) / 120(49.0)	6(7.9) / 33(43.4) / 37(48.7)	6(7.8) / 31(40.3) / 40(51.9)	2(8.0) / 9(36.0) / 14(56.0)
**rs2227306 (TT/TC/CC)**	52(15.4) / 162(47.9) / 122(36.1)	34(13.8) / 121(49.2) / 91(37.0)	11(14.5) / 39(51.3) / 26(34.2)	14(18.2) / 35(45.5) / 28(36.4)	4(16.0) / 8 (32.0) / 13(52.0)
**rs2227564 (CC/CT/TT)**	226(66.9) / 101(29.9) / 9(2.7)	170(69.1) / 70(28.5) / 6(2.4)	51(67.1) / 23(30.3) / 2(2.6)	51(66.2) / 25(32.5) / 1(1.3)	17(68.0) / 7(28.0) / 1(4.0)
**rs2228145 (CC/CA/AA)**	52(15.4) / 168 (49.7) / 116 (34.3)	34(13.8) / 119(48.4) / 93(37.8)	10(13.2) / 39(51.3) / 27(35.5)	12(15.6) / 44(57.1) / 21(27.3)	8(32.0) / 15(60) / 2(8.0)
**rs2228570 (AA/AG/GG)**	36(10.7) / 147(43.5) / 152 (45.0)	27(11.0) / 106(43.4) / 112(45.7)	6(7.9) / 34(45.3) / 35(46.7)	10(13.0) / 34(44.2) / 33(42.9)	1(4.0) / 14(56.0) / 10(40.0)
**rs2282679 (GG/GT/TT)**	28(8.3) / 133(39.3) / 175 (51.8)	22(8.9) / 103(41.9) / 121(49.2)	7(9.2) / 29(38.2) / 40(52.6)	9(11.7) / 30(39.0) / 38(49.4)	1(4.8) / 8(32.0) / 16(64.0)
**rs2430561 (TT/TA/AA)**	91 (26.9) / 161 (47.6) / 84 (24.9)	65(26.5) / 123(50.0) / 58(23.6)	20(26.3) / 37(48.7) / 19(25.0)	20(26.0) / 32(41.6) / 25(32.5)	5(20.0) / 12(48.0) / 8(32.0)

**rs2735940 (GG/GA/AA)**	74 (21.9) / 169(50.0) / 92(27.2)	55(22.4) / 121(49.4) / 69(28.2)	16(21.1) / 41(53.9) / 19(25.0)	14(18.2) / 40(51.9) / 23(29.9)	5(20.0) / 15(60.0) / 5(20.0)
**rs2794521 (TT/TC/CC)**	181(53.6) / 136(40.2) / 19(5.6)	131(53.3) / 104(42.3) / 11(4.5)	47(61.8) / 24(31.6) / 5(6.6)	43(55.8) / 29(37.7) / 5(6.5)	13(52.0) / 10(40.0) / 2(8.0)
**rs35697037 (GG/GA/AA)**	178(52.7) / 77(22.8) / 81(24.0)	128(52.0) / 53(21.5) / 65(26.4)	41(53.9) / 18(23.7) / 17(22.4)	42(54.5) / 16(20.8) / 19(24.7)	10(40.0) / 7(28.0) / 8(32.0)
**rs35705950 (GG/GT/TT)**	265(78.4) / 68(20.1) / 3(0.9)	191(77.6) / 53(21.5) / 2(0.8)	59(77.6) / 16(21.1) / 1(1.3)	59(76.6) / 18(23.4) / (-)	19(76.0) / 4(16.0) / 2(8.0)
**rs373740199 (CC/CT/TT)**	336(100) / (-) /(-)	246 (100) / (-) / (-)	76(100) / (-) / (-)	77(100) / (-) / (-)	25(100) / (-) / (-)
**rs386713 (CC/CT/TT)**	40(11.8) / 134(39.6) / 160(47.3)	28(11.5) / 94(38.5) / 122(50.0)	11(14.5) / 26(35.7) / 38(50.7)	8(10.4) / 34(44.2)/ 35(45.5)	3(12.0) / 11(44.0) / 11(44.0)
**rs398123017 (CC/CT/TT)**	336(100) / (-) /(-)	246 (100) / (-) / (-)	76(100) / (-) / (-)	77(100) / (-) / (-)	25(100) / (-) / (-)
**rs408265 (CC/CT/TT)**	155(45.9) / 136(40.2) / 45(13.3)	117(47.2) / 95(38.6) / 34(13.8)	37(48.7) / 29(38.2) / 10(13.2)	33(42.9) / 36(46.8) / 8(10.4)	11(44.0) / 8(32.0) / 6(24.0)
**rs419598 (TT/TC/CC)**	175(51.8) / 141(41.7) / 20(5.9)	127(51.6) / 104(42.3) / 15(6.1)	39(51.3) / 28(36.8) / 9(11.8)	42(54.5) / 33(42.9) / 2(2.6)	15(60.0) / 9(36.0) / 1(4.0)
**rs4588 (GG/GT/TT)**	173(51.2) / 132(39.1) / 28(8.3)	120(49.2) / 102(41.8) / 22(9.0)	40(52.6) / 29(38.2) / 7(9.2)	37(48.7) / 30(39.5) / 9(11.8)	16(64.0) / 8(32.0) / 1(4.0)
**rs5743890 (CC/CT/TT)**	10(3.0) / 94 (27.8) / 232 (68.6)	7(2.8) / 68(27.6) / 171(69.5)	4(5.3) / 21(27.6) / 51(67.1)	1(1.3) / 17(22.1) / 59(76.6)	(-) / 7(28.0) / 18(72.0)
**rs5743894 (TT/TC/CC)**	245(72.5) / 83(24.6) / 8(2.4)	186(75.6) / 56(22.8) / 4(1.6)	49(66.2) / 25(32.9) / 2(2.6)	55(71.4) / 21(27.3) / 1(1.3)	18(72.0) / 5(20.0) / 2(8.0)
**rs7041 (CC/CA/AA)**	114(33.7) / 172(50.9) / 50(14.8)	72(29.3) / 134(54.5) / 40(16.3)	27(35.5) / 35(46.1) / 14(18.4)	22(28.6) / 45(58.4) / 10(13.0)	11(44.0) / 12(48.0) / 2(8.0)
**rs727503468 (CC/CT/TT)**	336(100) / (-) /(-)	246 (100) / (-) / (-)	76(100) / (-) / (-)	77(100) / (-) / (-)	25(100) / (-) / (-)
**rs760506977 (CC/CT/TT)**	336(100) / (-) /(-)	246 (100) / (-) / (-)	76(100) / (-) / (-)	77(100) / (-) / (-)	25(100) / (-) / (-)
**rs876661305 (GG/GA/AA)**	336(100) / (-) /(-)	246 (100) / (-) / (-)	76(100) / (-) / (-)	77(100) / (-) / (-)	25(100) / (-) / (-)
